# Plasma Zinc and Magnesium Levels in Sickle Cell Disease Patients in Latakia, Syria

**DOI:** 10.7759/cureus.83526

**Published:** 2025-05-05

**Authors:** Zenab M Yousef, Remal A Asaad, Muhammed Imad M Khayat, Suzanne A Alshemali

**Affiliations:** 1 Department of Microbiology and Biochemistry, Tishreen University, Latakia, SYR; 2 Department of Laboratory Medicine, Tishreen Hospital, Latakia, SYR

**Keywords:** colorimetric, deficiency, magnesium, sickle cell disease, zinc

## Abstract

Background

Sickle cell disease (SCD) is a serious inherited disorder that affects millions of people worldwide. Zinc and magnesium are essential micronutrients involved in many cellular processes. Several studies have found that their deficiencies are common in SCD patients and may further complicate the disease. This study was conducted to examine plasma levels of zinc and magnesium in a group of SCD patients in Latakia.

Methods

A total of 85 SCD patients (52 males and 33 females) with both sickle cell anemia (HbSS) and hemoglobin sickle-beta-thalassemia (Hb S/β-Thal) genotypes at the steady state, and 30 healthy controls, were enrolled in this cross-sectional study with no age limits. Plasma zinc and magnesium levels were measured using colorimetric methods.

Results

Plasma zinc and magnesium levels were significantly lower in SCD patients compared to the controls (P < 0.05). Twelve SCD patients (14.1%) were zinc-deficient, and 37 (43%) had magnesium deficiency. Plasma zinc and magnesium levels were higher in HbSS patients than those with Hb S/β-Thal patients, but with no statistical significance (P > 0.05). Notably, all zinc-deficient SCD patients were < 16 years old. There was no significant (P > 0.05) difference in zinc deficiency distribution between males and females. Age and gender had no significant statistical relations with magnesium deficiency in SCD patients (P > 0.05).

Conclusion

This study has shown that plasma zinc and magnesium levels were significantly lower in SCD patients compared to the controls, with no significant difference between HbSS and Hb S/β-Thal genotypes. Zinc and magnesium administration may be required in SCD patients from an early age, especially zinc, to mitigate the adverse effects of their deficiencies.

## Introduction

Sickle cell disease (SCD) is the most common inherited hemoglobinopathy worldwide. It results from a single base-pair point mutation that leads to the substitution of glutamic acid with valine in the β-globin chain [1]. The hydrophobic valine predisposes sickle hemoglobin (HbS) to polymerize under low physiologic oxygen tension [2]. In time, this can lead to the formation of long polymers of HbS molecules that deform the red blood cells (RBCs) into the famous sickle shape [3]. Within the sphere of SCD, many subgroups exist, mainly sickle cell anemia (HbSS), hemoglobin SC disease (HbSC), and hemoglobin sickle-beta-thalassemia (Hb S/β-Thal), (β+ thalassemia or β0 thalassemia) [1]. The highest prevalence of SCD is among people of Sub-Saharan Africa, South Asia, the Middle East, and the Mediterranean [4]. It is estimated that over 300,000 infants are born annually with SCD around the world [5]. SCD is characterized by chronic hemolytic anemia, vaso-occlusion crisis, which manifests mainly in acute chest syndrome and severe acute and chronic pain crisis, as well as end-organ damage [4]. As a result of the high-energy expenditure associated with the accelerated rate of red blood cell turnover, it has been reported that SCD patients are at risk of many micronutrient deficiencies, including zinc and magnesium [6].

Second only to iron in its concentration in the human body, zinc is an essential micronutrient involved in many critical cellular processes such as protein synthesis and nucleic acid metabolism [7]. It has also become known that zinc is crucial for the normal development and function of immune cells, as well as for body growth [8]. Moreover, zinc has an antisickling effect due to its ability to increase hemoglobin affinity for oxygen [9], to antagonize calcium binding to red cell membrane [10], and by being a potent antioxidant [7]. Reports on zinc deficiency in sickle cell patients date back to the early 1970s [11], and it has since been linked to various complications of the disease [12]. These findings have been the impetus for several clinical trials that assessed the beneficial effects of zinc administration in sickle cell patients [13].

Magnesium is the second most abundant intracellular cation [14]. It is involved as a cofactor in more than 300 enzyme systems [15]. Several studies have reported low magnesium levels in patients with sickle cell disease [16]. Low intracellular magnesium is associated with elevated loss of KCl via the K-Cl cotransporter, promoting intracellular dehydration [17], and leading to RBC sickling [18].

Reports on zinc and magnesium levels in SCD patients in different countries are plentiful [6], but none have been published in Syria yet, a developing Mediterranean country where SCD is still a huge burden and patients do not receive adequate food [19]. This study was conducted to examine plasma levels of zinc and magnesium in a group of SCD patients in the coastal city of Latakia.

## Materials and methods

Study design and participants

A total of 85 SCD patients (52 males and 33 females) at the steady state were enrolled in this cross-sectional study with both HbSS and Hb S/β-Thal genotypes, and 30 HbAA apparently healthy individuals (17 males and 13 females) were also included as controls. Patients of all ages were recruited at the Center of Thalassemia and SCD and the Hematology Department at Latakia University Hospital between October 2023 and August 2024. Patients who had a blood transfusion in the past three months, an infection or inflammation during the previous four weeks prior to the study, and those on zinc or magnesium-containing supplements were excluded. All participants or their parents provided informed consent, as approved by the Bioethics Committee at Latakia University (Approval No. 4310, dated 18/7/2023) before inclusion. Data relevant to our study (age, exact phenotype of the SCD) was extracted from patients’ medical records and through personal interviews.

Sample collection and laboratory analysis

Fasting venous blood samples were collected on heparin tubes and centrifuged at 3000 rpm for five minutes. Plasma was separated within one hour of collection and kept at -20°C pending analysis. Hemolyzed or highly lipemic samples were rejected. Zinc and magnesium levels were measured by colorimetric methods. For zinc, laboratory analysis was carried out on RIELE Photometer 5010, using MEDICHEM kit (Lot. No. 236502, Syria) with reference ranges provided by the manufacturer (Appendix). Plasma levels of magnesium were measured on MINDRAY BS-380, using the BioSystems kit (Lot. No. 45532, Spain) with a reference range of 1.7-2.4 mg/dl.

Statistical analysis

Data from the study were analyzed using the SPSS software, version 20 (IBM Corp., Armonk, NY) and presented as mean ± standard deviation (SD). The student’s t-test was used to compare means between two groups. The Chi-square test was used to compare the prevalence of zinc and magnesium deficiencies in SCD patients and control groups. P-value <0.05 was considered statistically significant.

## Results

Demographic characteristics of the study participants

Our study included 115 participants, 85 SCD patients (49 with HbSS and 36 with Hb S/β-Thal) and 30 healthy subjects as controls. Participants ranged in age from 2.5 to 42 (13.6 ± 7.4) and 2 to 27 (13.1 ± 7.7) years for SCD patients and controls, respectively. The majority of HbSS patients 27 (55.1%), Hb S/β-Thal patients 27 (75%), and controls 19 (63.3%) were under 16 years old. Percentages of males were higher than those of females in all study groups (Table 1). No significant differences in age and gender distribution between the study groups were found (P > 0.05). Demographic characteristics of participants are displayed in Table 1.

**Table 1:** Demographic characteristics of the study participants SCD: Sickle cell disease, HbSS: Hemoglobin SS, Hb S/β-Thal: Hemoglobin S/β-Thalassemia.

No. (%)		SCD patients (n=85)		Controls (n=30)	P-value
		HbSS (n=49)	Hb S/β-Thal (n=36)		
Age (years)	<16	27 (55.1%)	27 (75%)	19 (63.3%)	0.170
	≥16	22 (44.9%)	9 (25%)	11 (36.7%)	
Gender	Male	31 (63.3%)	21 (58.3%)	17 (56.7%)	0.819
	Female	18 (36.7%)	15 (41.7%)	13 (43.3%)	

Mean plasma zinc and magnesium levels in SCD patients and controls

**Figure 1:**
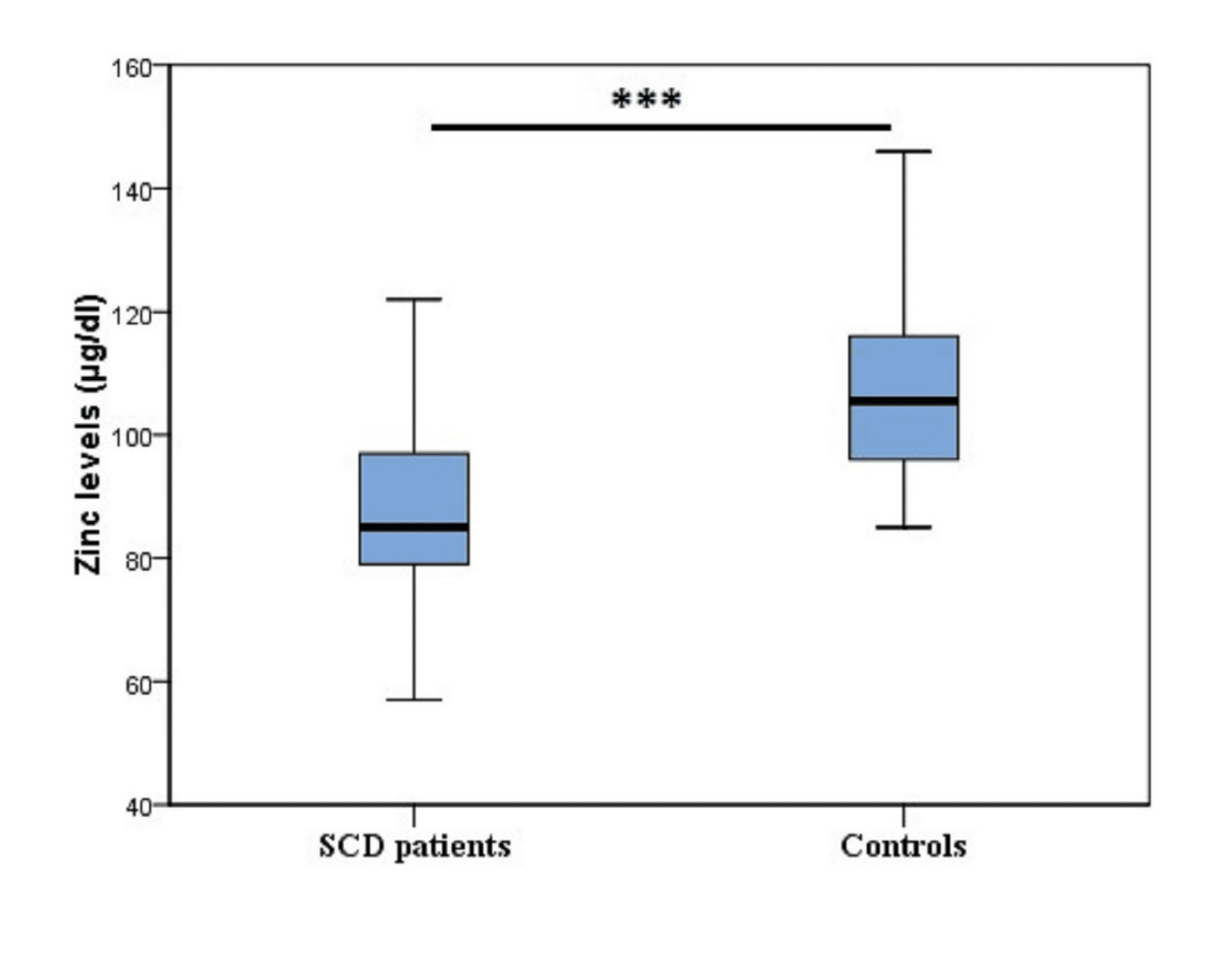
Plasma zinc levels in the study groups The X-axis shows the Box Plots of plasma zinc levels of sickle cell disease (SCD) patients and controls; the Y-axis is the actual recorded zinc levels along the Box Plot. For SCD patients: Median: 85 µg/dl; Interquartile range (IQR): 19 µg/dl; the whiskers extend from 57 to 124 µg/dl. For Controls: Median: 105.5 µg/dl; Interquartile range (IQR): 20 µg/dl; the whiskers extend from 85 to 146 µg/dl; *** P <0.001.

**Figure 2:**
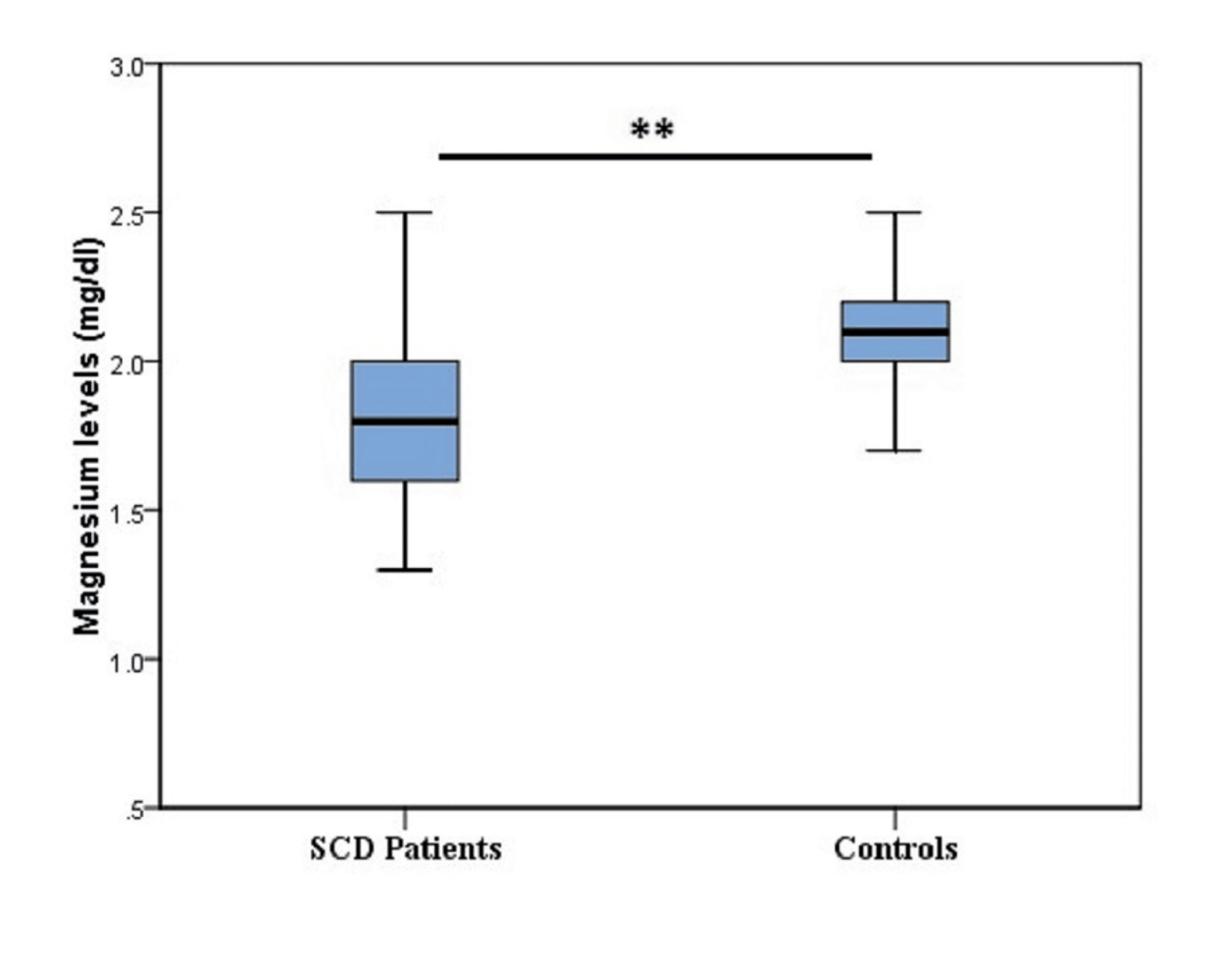
Plasma magnesium levels in the study groups. The X-axis shows the Box Plots of plasma magnesium levels of sickle cell disease (SCD) patients and controls; the Y-axis is the actual recorded magnesium levels along the Box Plot. For SCD patients: Median: 1.8 mg/dl; Interquartile range (IQR): 0.4 mg/dl; the whiskers extend from 1.3 to 2.5 mg/dl. For Controls: Median: 2.1 mg/dl; Interquartile range (IQR): 0.2 mg/dl; the whiskers extend from 1.7 to 2.5 mg/dl; ** P <0.01

The average plasma zinc levels among SCD patients (88.5 ± 15.2 µg/dl) were significantly lower than those in controls (106.9 ± 14.1 µg/dl; P = 0.000) (Figure 1). Likewise, mean plasma levels of magnesium were also significantly higher in the control group (2.1 ± 0.2 mg/dl) compared to the SCD patients (1.9 ± 0.4 mg/dl; P = 0.003) (Figure 2).

Mean plasma zinc and magnesium levels in HbSS and Hb S/β-Thal subgroups

Plasma zinc levels were higher (89.4 ± 16.5 µg/dl) in HbSS patients than in Hb S/β-Thal patients (87.3 ± 13.3 µg/dl), but with no statistical significance. A similar finding was observed when plasma magnesium levels between the two subgroups were compared (P = 0.171) (Table 2).

**Table 2:** Plasma zinc and magnesium levels of HbSS and Hb S/β-Thal patients HbSS: Hemoglobin SS, Hb S/β-Thal: Hemoglobin S/β-Thalassemia.

Variable	HbSS (n=49)	Hb S/β-Thal (n=36)	P-value
Zinc (µg/dl)	89.4 ± 16.5	87.3 ± 13.3	0.514
Magnesium (mg/dl)	1.9 ± 0.4	1.8 ± 0.4	0.171

Prevalence of zinc and magnesium deficiency in the study groups

**Figure 3:**
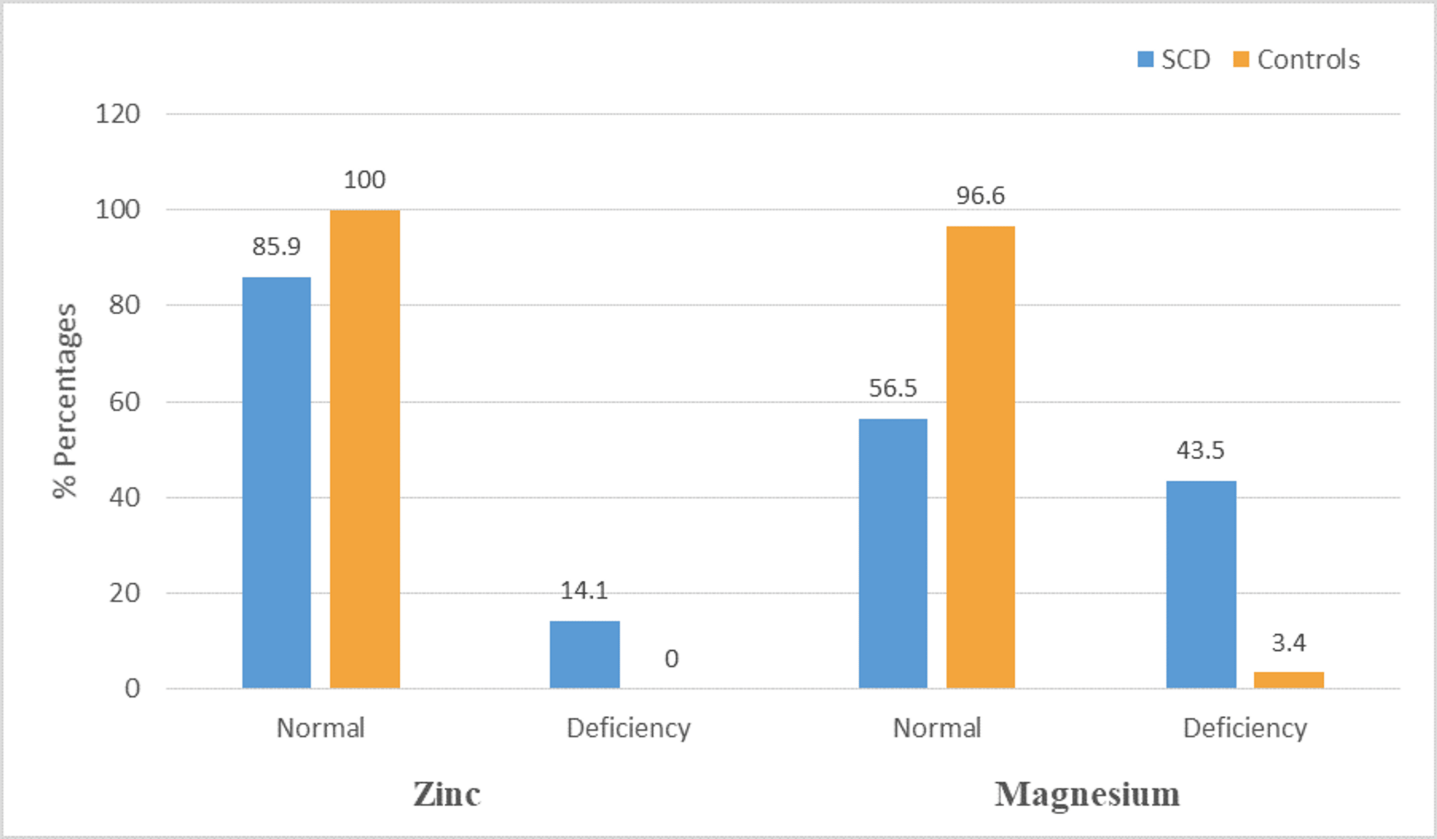
Prevalence of zinc and magnesium deficiency in study groups SCD: Sickle cell disease.

Twelve (14.1%) of SCD patients were zinc deficient, compared to none of the controls (P = 0.034). Meanwhile, 37 (43.5%) were deficient in magnesium, compared to only one case (3.4%) in the controls (P = 0.000). The prevalence of zinc and magnesium deficiencies in study participants is displayed in Figure 3.

Deficiency distribution of zinc and magnesium in SCD patients according to age and gender

Age distribution of zinc deficiency showed that 12 (100%) of zinc-deficient patients were < 16 years old. There was no significant (P > 0.05) difference in zinc deficiency distribution between males and females. Age and gender had no significant statistical relations with magnesium deficiency in SCD patients (Table 3).

**Table 3:** Zinc and magnesium deficiency according to age and gender in the study groups

No. (%)		SCD patients (n=85)			
		Zinc	P-value	Magnesium	P-value
Deficiency by age (years)	<16	12 (100%)	0.003	24 (64.9%)	1.000
	≥16	0 (0%)		13 (35.1%)	
Deficiency by gender	Male	8 (66.7%)	0.759	19 (51.4%)	0.120
	Female	4 (33.3%)		18 (48.6%)	

## Discussion

SCD is a serious disease that affects millions of people worldwide, with many complications that are associated with high rates of morbidity and mortality. SCD is characterized by chronic hemolysis, vaso-occlusive pain crisis, and oxidative stress [4]. Several studies have indicated that micronutrient deficiencies, including zinc and magnesium, were common in patients with SCD, further worsening patients' quality of life through their impact on disease complications such as increased vaso-occlusive pain crises. This led to several interventional trials to assess the benefits of zinc and magnesium administration in SCD patients [13]. No published reports from Syria about the levels of zinc and magnesium in SCD patients are available, although many hematologists prescribe zinc supplements to their SCD patients (personal communications). This study included 115 participants (52 males vs 33 females, 49 HbSS patients vs 36 Hb S/β-Thal patients) regardless of age, and 30 healthy controls. All SCD patients were in steady state, defined by the absence of an acute painful episode, an infection or inflammation for at least four weeks, and no history of blood transfusion in the last three months. All but two have been taking hydroxyurea for different periods of time.

Chronic hemolysis and increased urinary loss due to abnormal renal tubular reabsorption are well-known predisposing factors to hypozincemia in SCD patients [20]. In our study, we found that mean plasma levels of zinc were significantly lower in SCD patients compared to the controls (88.5 vs 106.9 µg/dl, P = 0.000), a finding that is similar to numerous studies [10,21]. On the other hand, a study in the United States found no significant difference in plasma zinc levels between SCD patients and healthy controls [22]. This could be due to variations in analytic methods and different ethnic backgrounds. While none of the subjects in the control group had zinc deficiency, probably due to the small sample size, 12 (14.1%) of our SCD patients had varying degrees of hypozincemia. This percentage is lower than those reported by several studies [23,24]. For example, Leonard et al. found that 44% of SCD children had zinc deficiency [23]. These different rates may be attributable to variations in analytical techniques, adoption of uncommon cut-offs, ethnicity, socioeconomic factors, and the different demographic characteristics of studied patients. Plasma zinc levels showed no significant differences between the HbSS and Hb S/β-Thal subgroups, which may indicate similarities in their underlying pathophysiological conditions. Furthermore, gender was not significantly associated with zinc deficiency among our SCD patients (Table 3), a finding that aligns with results from a Nigerian study [25]. Notably, we found that all zinc-deficient patients were < 16 years. The higher zinc requirements in children and adolescents may explain this finding. A study conducted in Congo reported no difference in the frequency of zinc deficiency between children and adults; this could be again explained by differences in the adopted cut-offs and different socioeconomic factors [26].

High levels of magnesium have been shown to inhibit the K-Cl cotransporter system [27], which is highly activated in sickle cells [17]. Mean plasma levels of magnesium in our study were significantly lower in SCD patients compared to the controls, as shown in Figure 2. This finding may be due to urinary magnesium loss, chronic hemolysis, and increased magnesium consumption to inhibit the K-Cl cotransporter system. Our findings were in line with several studies [16,28]. Nevertheless, it contrasted with a study in Nigeria [29], which found no significant difference in mean serum magnesium levels between SCD patients and controls. We also found that magnesium deficiency was present in 37 (43.5%) SCD patients, compared to only one (3.4%) of the controls. These results are consistent with a study from Ghana, which reported magnesium deficiency in 39.2% of SCD patients and 4.2% of controls [16]. In our study, there was no significant difference in mean plasma magnesium levels between the HbSS and Hb S/β-Thal subgroups, a finding that aligns with results from a study conducted in the United States [28]. Additionally, magnesium deficiency among SCD patients showed no significant association with age or gender, which is in agreement with observations reported by a research group in Iraq [30].

There are four major limitations in our study that could be addressed in future research. First, there is a small sample size; second, we were unable to determine the zinc content of the last meal due to the lack of accurate responses from patients. Third, due to incomplete documentation in some medical records, part of the information used to determine the disease’s steady state was based on patients’ self-reports. Fourth, we used the colorimetric method due to the limited availability of more accurate analytical methods such as atomic absorption spectrometry in Syria.

## Conclusions

Our study has shown that plasma zinc and magnesium levels were significantly lower in SCD patients compared to controls, with no difference between HbSS and HbS/β-Thal genotypes. All our zinc-deficient SCD patients were under 16 years old. We recommend conducting larger studies in both children and adults to validate our findings and examine their association with the disease complications. SCD has been associated with delayed psychomotor development, which is also linked to zinc deficiency. We also recommend examining the outcomes of administering zinc and magnesium supplements to SCD patients.

## Appendices

**Table 4:** Reference ranges of zinc levels by age and gender

Age		Zinc (µg/dl)	Zinc (µmol/l)
< 4 months		65 – 137	10 – 21
4-12 months		65 – 130	10 – 20
1-5 years		65 – 118	10 – 18
6-9 years		78 – 105	12 – 16
10-13 years	Male	78 – 98	12 – 15
	Female	78 – 118	12 – 18
14-19 years	Male	65 – 118	10 – 18
	Female	59 – 98	9 – 15
Adults		46 – 150	7 – 23
